# Therapeutic approaches in heart failure with preserved ejection fraction: past, present, and future

**DOI:** 10.1007/s00392-020-01633-w

**Published:** 2020-03-31

**Authors:** Jan Wintrich, Ingrid Kindermann, Christian Ukena, Simina Selejan, Christian Werner, Christoph Maack, Ulrich Laufs, Carsten Tschöpe, Stefan D. Anker, Carolyn S. P. Lam, Adriaan A. Voors, Michael Böhm

**Affiliations:** 1 Klinik für Innere Medizin III-Kardiologie, Angiologie und Internistische Intensivmedizin, Universitätsklinikum des Saarlandes und Medizinische Fakultät der Universität des Saarlandes, Kirrberger Straße, 66421 Homburg/Saar, Germany; 2 Comprehensive Heart Failure Center (CHFC), University Clinic Würzburg, Würzburg, Germany; 3 Klinik und Poliklinik für Kardiologie im Department für Innere Medizin, Neurologie und Dermatologie, Universitätsklinikum Leipzig, Leipzig, Germany; 4 Department of Cardiology, Universitätsmedizin Berlin, Charite, Campus Rudolf Virchow Clinic (CVK), Augustenburger Platz 1, 13353 Berlin, Germany; 5 German Center for Cardiovascular Research (DZHK), Partner Site, Berlin, Germany; 6 Berlin-Brandenburg Institute of Health/Center for Regenerative Therapies (BIHCRT), Berlin, Germany; 7 National Heart Centre, Singapore and Duke-National University of Singapore, Singapore, Singapore; 8 University Medical Centre Groningen, Groningen, The Netherlands; 9 The George Institute for Global Health, Sydney, Australia

**Keywords:** Heart failure, Preserved ejection fraction, Pharmacotherapy in HFpEF, LCZ696, Device therapy

## Abstract

In contrast to the wealth of proven therapies for heart failure with reduced ejection fraction (HFrEF), therapeutic efforts in the past have failed to improve outcomes in heart failure with preserved ejection fraction (HFpEF). Moreover, to this day, diagnosis of HFpEF remains controversial. However, there is growing appreciation that HFpEF represents a heterogeneous syndrome with various phenotypes and comorbidities which are hardly to differentiate solely by LVEF and might benefit from individually tailored approaches. These hypotheses are supported by the recently presented PARAGON-HF trial. Although treatment with LCZ696 did not result in a significantly lower rate of total hospitalizations for heart failure and death from cardiovascular causes among HFpEF patients, subanalyses suggest beneficial effects in female patients and those with an LVEF between 45 and 57%. In the future, prospective randomized trials should focus on dedicated, well-defined subgroups based on various information such as clinical characteristics, biomarker levels, and imaging modalities. These could clarify the role of LCZ696 in selected individuals. Furthermore, sodium-glucose cotransporter-2 inhibitors have just proven efficient in HFrEF patients and are currently also studied in large prospective clinical trials enrolling HFpEF patients. In addition, several novel disease-modifying drugs that pursue different strategies such as targeting cardiac inflammation and fibrosis have delivered preliminary optimistic results and are subject of further research. Moreover, innovative device therapies may enhance management of HFpEF, but need prospective adequately powered clinical trials to confirm safety and efficacy regarding clinical outcomes. This review highlights the past, present, and future therapeutic approaches in HFpEF.

## Introduction

Heart failure (HF) poses a growing burden for health systems worldwide as incidence and prevalence is rising annually. Typically, the term HF was applied to patients with reduced ejection fraction only, until the first reports on patients suffering from symptoms of HF despite having normal left-ventricular ejection fraction (LVEF) and small hearts emerged [1–3]. Initially, the condition was referred to as “diastolic heart failure” according to the different appearance compared to “systolic heart failure”. However, this has led to discussions among the scientific community, since a clear differentiation between systolic and diastolic dysfunction is rather hypothetical than physiological [4]. It was even shown that severity of diastolic dysfunction may be greater in patients with impairment of systolic function than in those without [5] and that systolic dysfunction can also be detected in patients with preserved ejection fraction [6]. Therefore, the European Society of Cardiology (ESC) focused on objective findings and proposed the term “heart failure with preserved ejection fraction” (HFpEF). In the latest 2016 guidelines, HF is differentiated in three different forms depending on LVEF: HFpEF (LVEF ≥ 50%), HFrEF (“heart failure with reduced ejection fraction”, LVEF < 40%), and HFmEF (“heart failure with mid-range ejection fraction”, LVEF > ≥ 40 and < 50%) [7]. In contrast to the latest advances in therapy of HFrEF, HFpEF remains a challenge, in which many established HF drugs have failed to improve prognosis. This review highlights the main epidemiological and pathophysiological aspects in HFpEF and discusses dilemmas in management of HFpEF as well as promising therapeutic options for the future.

## Dilemma in diagnosing HFpEF

HFpEF mostly affects older patients, predominantly females. Depending on various factors (e.g., definition and time of publication), the proportion of HFpEF among HF patients ranges from 22 to 73% [7]. Patients with HFpEF are a heterogeneous group with numerous underlying aetiologies and pathophysiological abnormalities [7]. Thus, diagnosis of HFpEF can be challenging, as it rather describes a clinical syndrome than a single clinical diagnosis [8]. Also, there have been debates whether the definition of HFpEF should be based solely on LVEF, since LVEF-based HF subgroups may exhibit significantly overlapping phenotypes [9]. This issue has resulted in proposition of diagnostic algorithms which take various diagnostic measures such as clinical characteristics, laboratory and echocardiographic findings, as well as sophisticated imaging modalities and invasive haemodynamic measurements into account. For instance, a composite HFpEF score determined by presence of atrial fibrillation, obesity, age > 60 years, treatment with ≥ 2 antihypertensives, echocardiographic *E*/*e*′ ratio > 9, and echocardiographic pulmonary artery systolic pressure > 35 mmHg has been shown to substantially identify patients at high risk of HFpEF that should undergo further evaluation [10]. According to the updated consensus recommendation by the Heart Failure Association (HFA) of the ESC [8], a step-wise diagnostic process should be applied in patients with suspected HFpEF. After an initial work-up based on clinical parameters and non-invasive tests (e.g. ECG, echocardiography, blood tests), the authors suggest a risk stratification by using the ‘HFA-PEFF’ score in selected patients. In this score, patients are stratified in three different groups (low risk, intermediate risk, and high risk) according to echocardiographic parameters and biomarker levels. While patients identified as high risk should be diagnosed with HFpEF, patients at intermediate risk should undergo echo stress tests or if inconclusive, invasive haemodynamic measurements, to establish the diagnosis of HFpEF. Finally, the authors recommend an aetiological work-up which includes ergometry, blood tests, genetic testing, imaging modalities (particularly cardiac magnetic resonance imaging), and, in rare cases, myocardial biopsy. This suggested exclusion of specific causes in the etiology of HFpEF, for example primary cardiomyopathies and storage diseases such as M. Fabry and amyloidosis, as well as pericardial diseases such as constrictive pericarditis, may be crucial for an individually tailored specific treatment of the HFpEF syndrome. For instance, initiation of tafamidis in transthyretin amyloid cardiomyopathy is of great importance, as these patients suffer from a poor prognosis [11]. If untreated, the median survival time of patients with a wild-type transthyretin amyloidosis is 3.6 years after diagnosis and 2.5 years with a hereditary transthyretin amyloidosis [12, 13]. The benzoxazole derivative tafamidis prevents amyloidogenesis by binding to the thyroxine-binding sites of transthyretin. In the recent Transthyretin Amyloidosis Cardiomyopathy Clinical Trial (ATTR-ACT) including 441 patients with transthyretin amyloid cardiomyopathy, therapy with tafamidis led to a significant reduction in all-cause mortality and rate of CV hospitalizations compared to placebo [14].

## Current understanding of pathophysiological mechanisms in HFpEF

**Fig. 1:**
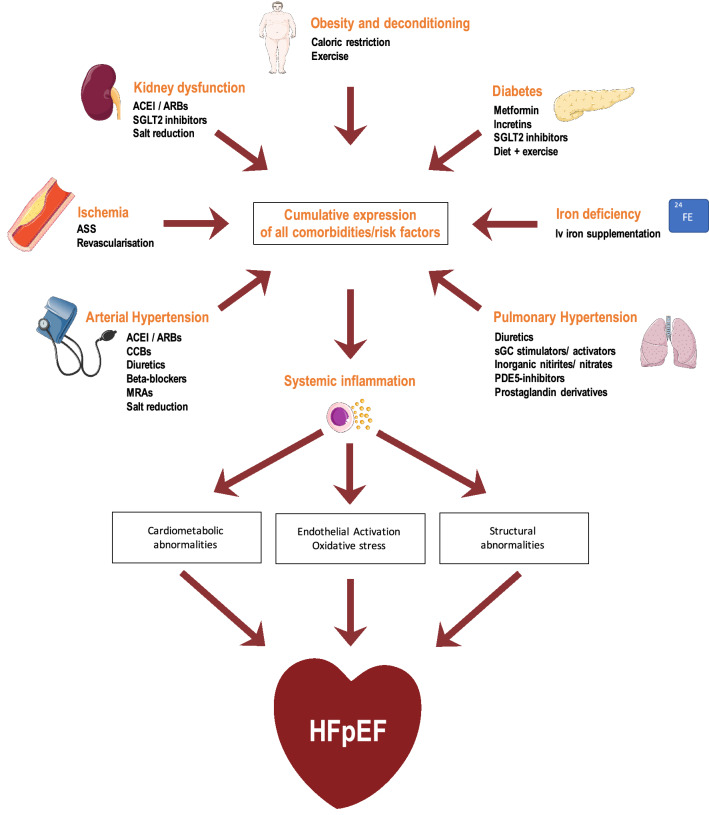
Current model on pathophysiology and management of comorbidities and risk factors in HFpEF. Cumulative expression of the shown comorbidities and risk factors can cause systemic inflammation which can then lead to development of HFpEF [2]. *ACEI* angiotensin-converting enzyme inhibitor, *ARB* angiotensin receptor blocker, *CCB* calcium channel blocker, *MRA* mineralocorticoid receptor antagonist, *PDE5* hosphodiesterase-5, *sCG* soluble guanylate cyclase, *SGLT2* sodium-glucose cotransporter-2. Figure modified according to Tschöpe et al. [4] and Lam et al. [9]

Currently, the precise pathophysiological processes in HFpEF are incompletely resolved, since animal models are sparse. This is due to a high prevalence of comorbidities in HFpEF patients, which is difficult to be translated into animal models, which are typically younger and less comorbid [4]. However, there is consensus that HFpEF is associated with systemic inflammation [15], which is triggered by the cumulative expression of various risk factors and comorbidities (Fig. 1). If no specific disease is the cause, the most common risk factors/comorbidities of HFpEF are age, female gender, renal impairment, diabetes, hypertension, as well as obesity and deconditioning [16]. Typically, in contrast to HFrEF patients, patients suffering from HFpEF are older, have a higher average body mass index, are more likely to be female, and exhibit a lower prevalence of ischemic heart disease [17]. Activation of the endothelium through the systemic inflammatory state eventually causes oxidative stress [18]. As a consequence, reactive oxygen species (ROS) directly react with nitric oxide (NO) and reduce its bioavailability. In addition, ROS may cause eNOS uncoupling which leads to production of highly reactive superoxide (O_2_^−^) instead of NO. These processes result in a vasoconstricting, pro-inflammatory, and pro-thrombotic state of endothelial dysfunction [19]. Furthermore, alterations of both the myocytic and non-myocytic compartment can increase diastolic stiffness and may contribute to development of HFpEF [20, 21]. For instance, reduction of NO bioavailability by oxidative stress and inflammatory cytokines downregulates the nitrogen monoxide–cyclic guanosine monophosphate–protein kinase G (NO–cGMP–PKG) pathway, and, therefore, decreases PKG activity. PKG plays an essential role in regulating phosphorylation, isoform switching, and oxidative modifications of the cytoskeletal protein titin, which mainly determines cardiomyocyte stiffness [22]. Besides cardiomyocyte stiffness, changes in the composition and structure of the non-myocytic compartment contribute to diastolic stiffness [19]. Endothelial dysfunction is associated with adherence and infiltration of monocytes and stimulation of integrated macrophages. By secretion of pro-fibrotic substances, in particular transforming growth factor β (TGF-β) [23], these cells promote myofibroblast differentiation and eventually collagen secretion, leading to extracellular fibrosis [24, 25]. In addition, galectin-3, a lectin-binding galactoside, has been suggested to be another major mediator of myocardial fibrosis in HFpEF, which enhances collagen secretion by binding to myofibroblasts and may be in part responsible for the conferral of the detrimental effects of aldosterone [26, 27]. Moreover, myocardial fibrosis in HFpEF can result from hypertension, aging, metabolic triggers, and infrequently reparative processes [28]. Finally, cardiometabolic functional abnormalities, e.g., abnormal mitochondrial structure and function, change in substrate utilization and intracellular calcium overload, are thought to be another important pathomechanism in HFpEF, although these assumptions are primarily derived from studies in HFrEF [29].

## Treatment of HFpEF

### Focus on comorbidities

Clinical findings suggest that prognosis in patients with HFpEF is highly influenced by comorbidities [30–32]. This concept is addressed in the OPTIMIZE-HFpEF trial (NCT02425371). Thus, adequate treatment of comorbidities in HFpEF might be of crucial importance and patients should be regularly screened for these conditions [33] (Fig. 1). For instance, obesity and deconditioning are common risk factors in HFpEF. In a sub-analysis of the I-PRESERVE trial, 71% of all 4109 patients had a body mass index ≥ 26.5 kg/m^2^ and 21% had a BMI between 23.5 and 26.4% kg/m^2^ [34]. Moreover, the risk for the primary endpoint (death from any cause or hospitalization for a CV cause, that is, HF, myocardial infarction, unstable angina, arrhythmia, or stroke) was increased in patients with BMI < 23.5 kg/m^2^ and in those with BMI ≥ 35 kg/m^2^. Both physical activity (PA) and caloric restriction are important non-pharmacological approaches to reduce obesity and deconditioning and have shown to be associated with prognostic effects. In a post hoc analysis of the TOPCAT trial, risk of HF hospitalization and mortality was lower in physically high-active HFpEF patients than in intermediate-active and poorly active patients [35]. In the prospective Ex-DHF pilot trial, supervised exercise training (ET) improved exercise capacity and QOL and led to atrial reverse remodeling and reduction of diastolic dysfunction in HFpEF patients [36]. The ongoing Ex-DHF trial aims to evaluate long-term effects of supervised ET on a total of 320 patients [37]. Furthermore, prescription of a 20-week hypocaloric diet was associated with an increased peak *V*O_2_ in a cohort of 100 obese HFpEF patients, most of which were female (81%). In addition, the effects were even greater when patients also had to join supervised exercise sessions three times a week, suggesting the combination of PA and diet to provide additive effects [38]. Another important comorbidity in HF patients is anemia due to iron deficiency [7]. In a small study with 190 symptomatic HFpEF patients, iron deficiency was present in 58.4% of all patients, while only 54 patients showed a corresponding anemia [39]. Interestingly, iron deficiency was significantly more prevalent in patients with severe diastolic dysfunction, and was associated with reduced exercise capacity and quality of life (QOL). Intravenously administered iron improves symptoms and QOL in patients with HFrEF [40]. Enhancing mitochondrial energy supply by iron supplementation has been discussed as one underlying mechanism, but whether this affects cardiac and/or skeletal muscles is currently unclear [41, 42]. Two current randomized-controlled trials (RCTs) (FAIR-HFpEF, PREFER-HF) focus on the effects of intravenously administered iron primarily on functional capacity in terms of six-minute walking distance (6MWD) as well as morbidity and mortality in HFpEF patients (NCT03074591, NCT03833336). Moreover, hypertension can cause recurring hospitalizations in HFpEF [43] and needs to be treated in accordance to the current hypertension guidelines [44]. Myocardial ischemia has also been frequently reported in HFpEF patients, contributing to greater deterioration in ventricular function and increased mortality [45]. Therefore, special emphasis should also be placed on adequate diagnostic measures and revascularization strategies. Additionally, atrial fibrillation (AF), the most common arrhythmia, often coexists with HFpEF [46]. According to a post hoc analysis of the TOPCAT trial, detection of AF represents an independent risk factor of adverse cardiovascular (CV) outcome (composite endpoint of CV mortality, aborted cardiac arrest, or HF hospitalization) [47]. While catheter ablation of AF leads to increased survival rates compared to antiarrhythmic drug therapy in HFrEF [48, 49], it is currently unclear if these effects equally account for HFpEF patients [50]. In a small retrospective analysis, effects of catheter ablation on symptom burden, NYHA functional class, in-hospital adverse event rate, and freedom from recurrent atrial arrhythmia at 12 months were similar in 97 HFrEF (LVEF < 50%) and 133 HFpEF (LVEF ≥ 50%) patients [51]. However, adequately powered, randomized trials are necessary, to assess the value of AF ablation in the collective of HFpEF patients.

### Dilemmas in past HFpEF trials

In past trials, there have been significant differences regarding the definition of HFpEF. In contrast to the ESC definition (LVEF ≥ 50%), major clinical trials such as the TOPCAT trial [52] or the recent PARAGON-trial [53] have included patients with an LVEF ≥ 45%. However, as mentioned, there are increasing concerns about defining HFpEF by LVEF only [9]. Furthermore, it is essential to acknowledge HFpEF as a heterogeneous syndrome most likely comprising various pathophysiological phenotypes which might need to be treated differently. Therefore, future clinical trials should focus on dedicated, well-defined patient cohorts which should not be solely based on LVEF.

### Conventional HF drugs in HFpEF

#### ACE inhibitors and AT1 antagonists

Stimulation of AT1 receptors induces myocardial hypertrophy and fibrosis which can then lead to HF [54]. ACE inhibitors and angiotensin II receptor blockers (ARBs), which target the renin–angiotensin–aldosterone system (RAAS) pathway and inhibit the activation of AT1 receptors, reduce morbidity and mortality in patients with HFrEF [55–57]. In patients with HFpEF, however, they have failed to improve clinical outcomes. In the I-PRESERVE trial, irbesartan did not reduce hospitalization rates for CV causes or all-cause mortality in patients with HF and LVEF of at least 45% [58]. In the CHARM-PRESERVED study, candesartan reduced HF hospitalizations, but not CV death rates [59]. Perindopril has been shown to improve symptoms and exercise capacity but not morbidity or mortality in 850 elderly patients with a mean age of 76 years (PEP-CHF) [60]. The VALIDD study compared effects of valsartan to other antihypertensive agents in patients with evidence of diastolic dysfunction and hypertension [61]. In both groups, diastolic function improved after reduction of blood pressure, regardless of the antihypertensive treatment.

#### Mineralocorticoid receptor antagonists

Mineralocorticoid receptor antagonists (MRAs) prevent the maladaptive effects of aldosterone. Aldosterone mediates myocardial fibrosis [62], contributing to myocardial stiffness and filling abnormalities. The ALDO-DHF trial proved that spironolactone had a positive impact on diastolic function by reducing the *E*/*e*′-ratio and decreased left-ventricular (LV) hypertrophy and NT-proBNP levels [63]. Surprisingly, HF symptoms, exercise tolerance, and QOL have not been significantly affected by spironolactone. In the international, multicenter TOPCAT trial, spironolactone failed to significantly improve CV outcomes in 3445 HFpEF patients (LVEF ≥ 45%) [52]. However, these findings might have been biased by regional differences. As compared to patients enrolled in the US, Canada, Brazil, and Argentina (the Americas), patients enrolled in Russia and Georgia exhibited markedly lower clinical event rates [64] and their concentrations of canrenone, an active metabolite of spironolactone, were much more likely to be undetectable, suggesting higher rates of patients’ incompliance [65]. These aspects might explain why spironolactone was able to reduce risk of CV death and HF hospitalization in the American population, while this did not account for patients from Russia and Georgia [64]. Furthermore, treatment effects of spironolactone were influenced by LVEF and have reached significance at the lower end of the ejection fraction spectrum [66]. As a result, MRAs can now be considered to decrease hospitalizations in appropriately selected patients with HFpEF, according to the updated ACC/AHA/HFSA guidelines [67]. Critically, it needs to be outlined that the regional interaction analyses of the TOPCAT trial were post hoc, which can, therefore, only serve as hypothesis generating. In addition, the *p* value for the treatment-by-region-interaction was not significant (*p* = 0.12) [64]. Moreover, when making recommendations about HF therapy based on regional interaction analyses, this should be equally applied to all HF drugs. For instance, beta-blockers have not shown any beneficial effects in the US population [68, 69], but are still recommended as an essential part of HF therapy in the USA. Furthermore, the potential mistakes in Russia and Georgia implied by the mentioned post hoc analyses were only possible because of the trial organization which wanted to save money by including Russians and Georgians.

In the future, new studies such as the German prospective SPIRIT-HF trial (2017-000697-11) and the large registry-randomized clinical trial SPIRRIT-HF (NCT02901184) will reevaluate therapy with spironolactone in HFpEF patients. In SPIRIT-HF, particular emphasis will lie on patient characterization and selection. Novel MRAs, such as nonsteroidal aldosterone antagonists, will also be evaluated [70, 71].

#### Beta-blockers

High heart rate (HR) predicts poor outcome in patients with HFpEF and sinus rhythm, but does not apply for those in atrial fibrillation, as shown in a post hoc analysis of the I-PRESERVE trial [72]. These findings were supported by a sub-analysis of the CHART-2 study, in which elevated HR was associated with a higher CV mortality in HFpEF patients [73]. The MAGICC registry confirmed the prognostic association of HR in sinus rhythm, but not in atrial fibrillation in 2285 HFrEF and 974 HFpEF patients [74]. Thus, several studies investigated whether beta-blockers induce positive prognostic effects in patients with HFpEF by helping to reduce HR. In a pre-specified sub-analysis of the SENIORS trial, no significant differences were observed regarding the prognostic impact of nebivolol, a β_1_-selective beta-blocker, in patients with impaired and preserved LV function (separation in this trial was LVEF > 35%) [75]. In the ELANDD study, 6 month treatment with nebivolol led to a reduction in HR, while it had no effect on exercise capacity in terms of 6MWTD and peak oxygen consumption (*V*O_2_) in 116 HFpEF patients [76]. A large meta-analysis on the prognostic effects of beta-blockers in HFpEF showed a reduction in mortality by 21%, but results were mainly influenced by findings from observational cohort studies [77]. In the pooled analysis of RCTs only, use of beta-blockers was associated with a reduced risk of mortality but without reaching statistical significance. The OPTIMIZE-HF registry, on the other hand, did not find a relevant prognostic effect of beta-blocker treatment in patients with HFpEF [17]. However, both the mentioned meta-analysis [77] and the OPTIMIZE-HF registry [17] did not assess potential differences in therapeutic efficacy between the different sub-classes of beta-blockers. Perhaps, beneficial effects may be present in selected sub-classes of beta-blockers which would need to be evaluated in further trials.

#### Angiotensin receptor neprilysin inhibitor

The angiotensin receptor neprilysin inhibitor LCZ696, combining the two acting agents valsartan and sacubitril, has revolutionized treatment of HFrEF. By inhibition of neprilysin, sacubitril increases ANP-, BNP- and CNP-plasma levels [33]. These peptides can then activate guanylyl cyclase resulting in formation of cGMP. Moreover, natriuretic peptides help to prevent myocardial fibrosis and to lower blood pressure due to vasodilation and increased diuresis [33]. As discussed above, prognosis of patients with HFpEF is affected by comorbidities such as diabetes. A post hoc analysis of the PARADIGM-HF trial revealed that sacubitril enhances glycemic control and reduces the necessity of insulin treatment in HFrEF patients [78]. This could be a further beneficial effect in patients with HFpEF, where diabetes is thought to trigger the disease. The PARAGON-HF trial evaluated therapy with LCZ696, and enrolled 4822 patients with HF and LVEF ≥ 45% [53]. As recently presented, LCZ696 failed to reduce the primary composite endpoint of total hospitalizations for HF and CV death. However, prespecified subgroup analyses suggested positive effects of LCZ696 in female patients and those with an LVEF at or below the median of all enrolled patients (45–57%). Similarly, it was shown that treatment effects of LCZ696 are modified by LVEF, leading to the greatest benefits in patients with an LVEF of < 50% [79]. These findings are in accordance with several post hoc analyses of previous HF trials such as TOPCAT [66], CHARM [80], and a meta-analysis on beta-blocker effects in HF [81] that have shown positive treatment effects for patients exhibiting an LVEF of 40–49%. Of note, these patients have to be categorized as HFmEF according to the ESC guidelines [7]. Moreover, a recent post hoc analysis of PARAGON-HF documented a significant treatment effect of LCZ696 in women, while there were no significant effects in men [82]. Furthermore, an important limitation of the PARAGON-HF trial consists in the missing exclusion of specific causes such as Amyloidosis and M. Fabry which are resistant to treatment with LCZ696.

In conclusion, results from the PARAGON-HF trial support the heterogeneity of the HFpEF syndrome as well as the importance of an individually tailored approach in HFpEF therapy. In this context, identifying specific causes of HFpEF by an aetiological work-up is of great importance. Moreover, LCZ696 might be associated with beneficial effects in female patients and those with a LVEF between 45–57% which would include both HFmEF and HFpEF patients. This aspect may underline the limitations of subdividing HF phenotypes solely by LVEF. As the primary endpoint of PARAGON-HF was neutral, new prospective randomized studies in dedicated subgroups might scrutinize efficacy of LCZ696 in selected individuals.

#### Ivabradine

In a mouse model of HFpEF, established by diabetic mice (db/db), β-adrenergic receptor-independent reduction of HR with ivabradine, an inhibitor of the funny current, improved vascular stiffness, as well as systolic and diastolic function [83]. However, according to experimental data, this particular mouse model is not associated with marked structural remodeling of the heart [84]. In the EDIFY study, ivabradine reduced HR by 30%, but failed to improve *E*/*e*′ ratio, exercise tolerance, and NT-proBNP levels in HFpEF patients [85]. Apparently, the pathophysiological concept of prolonging diastole to improve diastolic function and prognosis cannot be applied to patients with HFpEF. A plausible explanation might be that chronotropic incompetence in HFpEF patients contributes to impaired exercise tolerance and ivabradine further reduces the exercise-induced increase in HR [86].

#### Cardiac glycosides

In the DIG trial, cardiac glycosides were able to decrease the risk for overall hospitalization and hospitalization due to worsening HF in patients with HFrEF and HFpEF (LVEF > 45%) [58]. On the contrary, there have been no significant differences between digoxin and placebo regarding overall and CV mortality [59]. As a result, cardiac glycosides can be considered as a potential treatment to control tachyarrhythmia in patients with HFpEF.

### New options in treatment of HFpEF

**Fig. 2:**
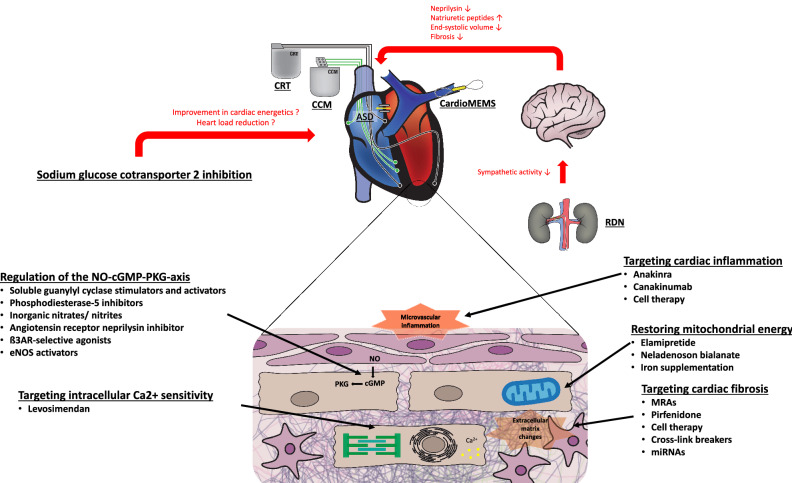
Main approaches regarding device and pharmacological therapy in HFpEF patients. Renal denervation can lower sympathetic activity resulting in decreased neprilysin activation, end-systolic volumes, and cardiac fibrosis as well as increased levels of natriuretic peptides. By implantation of an atrial shunt device, left-atrial pressure can be reduced. Continuous measurement of pulmonary artery pressure with the CardioMEMS device helps to prevent cardiac decompensation. CRT devices target mechanical LV dyssynchrony in HFpEF patients. CCM devices aim to enhance myocardial contractility. Main pharmacological approaches in HFpEF comprise regulation of the NO–cGMP–PKG-axis, restoring mitochondrial energy, modulation of intracellular Ca^2+^ sensitivity as well as targeting cardiac inflammation and fibrosis. Furthermore, inhibition of the sodium glucose cotransporter-2 represents another important approach in HFpEF therapy, although the exact pathomechanisms are currently unknown. *ASD* atrial shunt device, *CCM* cardiac contractility modulation, *CRT* cardiac resynchronization therapy, *eNOS* endothelial nitric oxide synthase, *miRNA* micro-RNA, *MRA* mineralocorticoid receptor antagonist, *NO–cGMP–PKG* nitrogen monoxide–cyclic guanosine monophosphate–protein kinase, *RDN* renal denervation. Figure modified according to Lam et al. [9] and Böhm et al. [135]

All main approaches regarding device and pharmacological therapy in HFpEF patients are highlighted in Fig. 2. Moreover, all current pharmacological and device trials in HFpEF patients are summarized in Tables 1, 2, 3.

**Table 1:** Current pharmacological and device trials in HFpEF patients focusing on clinical outcomes

Study name	Intervention	Study size	Primary endpoint	Identifier
*Sodium glucose cotransporter-2 inhibitors*				
EMPEROR-PRESERVED	Empagliflozin	4126	Change in CV death rate, time-to-first HF hospitalization	NCT03057951
DELIVER	Dapagliflozin	4700	Change in CV death rate, time-to-first HF hospitalization/first urgent HF visit	NCT03619213
SOLOIST	Sotagliflozin	4000	Change in CV death rate, time-to-first HF hospitalization	NCT03521934
*Mineralocorticoid receptor antagonists*				
SPIRRIT	Spironolactone	3500	Change in overall death rate	NCT02901184
SPIRIT-HF	Spironolactone	1300	Change in CV death rate, number of recurrent HF hospitalizations	2017-000697-11
*Device therapy*				
GUIDE-HF	CardioMEMS	3600	Change in all-cause mortality, total number of HF hospitalizations, iv diuretic visits	NCT03387813
REDUCE LAP-HF TRIAL II	IASD System II	608	Change in incidence of and time-to-CV mortality or first non-fatal, ischemic stroke, total rate per patient year of HF admissions or healthcare facility visits for IV diuresis for HF and time-to-first HF event, KCCQ	NCT03088033

*CV* cardiovascular, *HF* heart failure, *IV* intravenous, *KCCQ* Kansas City Cardiomyopathy Questionnaire, *QOL* quality of life

**Table 2:** Current pharmacological and device trials in HFpEF patients focusing on biomarker levels, quality of life, and cognitive function

Study name	Intervention	Study size	Primary endpoint	Identifier
*Soluble guanylyl cyclase stimulators and activators*				
SERENADE	Macitentan	300	Change in NT-proBNP levels	NCT03153111
VITALITY	Vericiguat	735	Change in QOL	NCT03547583
*Inorganic nitrates/nitrites*				
PMED	Oral nitrate	120	Change in nitrate/nitrite level, microbiome	NCT02980068
*Angiotensin receptor neprilysin inhibitor*				
PERSPECTIVE	LCZ696	520	Change in cognitive function	NCT02884206
PARALLAX	LCZ696	2500	Change in NT-proBNP levels	NCT03066804
*Sodium glucose cotransporter-2 inhibitors*				
PRESERVED-HF	Dapagliflozin	320	Change in NT-proBNP levels	NCT03030235
ERADICATE-HF	Ertugliflozin	36	Change in proximal sodium reabsorption	NCT03416270
*Restoring mitochondrial energy*				
Elamipretide in patients hospitalized with congestion due to HF	Elamipretide	300	Change in NT-pro-BNP levels	NCT02914665
*Device therapy*				
CCM-HFpEF	CCM	50	Change in QOL	NCT03240237

*QOL* quality of life, *NT-pro-BNP* N-terminal-pro hormone B-type natriuretic peptide

**Table 3:** Current pharmacological and device trials in HFpEF patients focusing on echo/hemodynamic parameters

Study name	Intervention	Study size	Primary endpoint	Identifier
*Soluble guanylyl cyclase stimulators and activators*				
CAPACITY-HF	IW 1973	184	Change in peak *V*O_2_	NCT03254485
DYNAMIC	Riociguat	114	Change in CO	NCT02744339
*Phosphodiesterase-5 inhibitors*				
Sildenafil in HFPEF and PH	Sildenafil	52	Change in PAP, CO	2010-020153-14
*Inorganic nitrates/nitrites*				
INABLE	Oral inorganic nitrite	100	Change in peak *V*O_2_	NCT0271312
KNO3CK OUT HFPEF	Oral potassium nitrate	76	Change in QOL, muscle blood flow, SVR reserve	NCT0284079
PH-HFPEF	Oral nitrite	26	Change in PAP at exercise	NCT03015402
ONOH	Oral nitrite	18	Change in peak *V*O_2_	NCT02918552
Neo40	Oral nitrate supplement	25	Change in exercise capacity, *E*/*e*′, RVSP	NCT03289481
*3AR-selective agonists*				
BETA3_LVH	Mirabegron	297	Change in LVMI, *E*/*e*′	NCT02599480
SPHERE-HF	Mirabegron	80	Change in PVR	NCT02775539
*Sodium glucose cotransporter-2 inhibitors*				
EMPERIAL-PRESERVED	Empagliflozin	300	Change in 6MWD	NCT03448406
*Other antidiabetic drugs*				
Metformin for PH + HFPEF	Metformin	32	Change in PAP at exercise	NCT03629340
cGETS	Sitagliptin	25	Change in hemodynamics during Dobutamine stress test, diastolic dysfunction, LV hypertrophy	2012-002877-71
*Pirfenidone*				
PIROUETTE	Pirfenidone	200	Change in ECV	NCT02932566
*Cell therapy*				
CELL-pEF	CD34^+^ cell therapy	30	Change in *E*/*e*′	NCT02923609
*Restoring mitochondrial energy*				
Elamipretide in subjects with stable HFpEF	Elamipretide	46	Change in *E*/*e*′	NCT02814097
FAIR-HFpEF	Ferric carboxymaltose	200 Change in 6MWD	NCT03074591	
PREFER-HF	Ferric carboxymaltose	72	Change in 6MWD	NCT03833336
*Targeting intracellular Ca*^*2+*^* sensitivity*				
HELP	Levosimendan	36	Change in PCWP at exercise	NCT03541603
*Prostaglandin derivatives*				
ILO-HOPE	Iloprost	34	Change in PCWP after exercise	NCT03620526
SOUTHPAW	Treprostinil	310	Change in 6MWD	NCT03037580
*Device therapy*				
RAPID-HF	CRT	30	Change in *V*O_2_ at VAT	NCT02145351
PREFECTUS	CRT	10	Change in systolic and diastolic reserve index	NCT03338374

*CO* cardiac output, *ECV* extracellular volume fraction, *LVMI* left-ventricular mass index, *PAP* pulmonary arterial pressure, *PCWP* pulmonary capillary wedge pressure, *PVR* pulmonary vascular resistance, *QOL* quality of life, *RVSP* right-ventricular systolic pressure, *SVR* systemic vascular resistance, *VAT* ventilatory anaerobic threshold, *VO*_*2*_ oxygen consumption

#### Pharmacological

##### Regulation of the NO–cGMP–PKG-axis

**Fig. 3:**
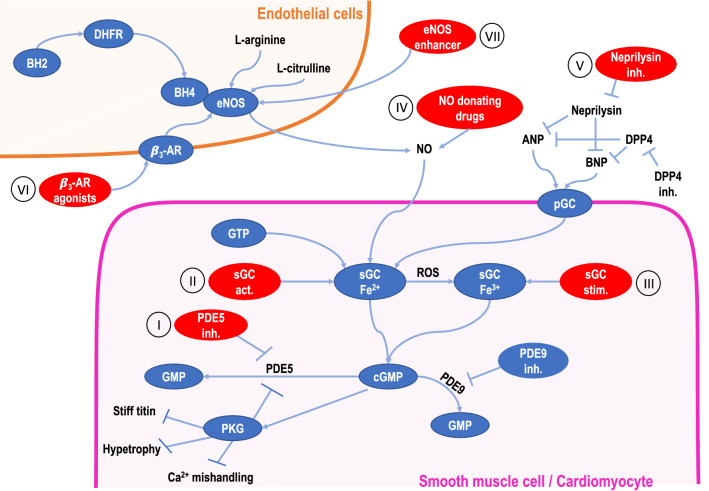
Current pharmacological approaches regarding regulation of the NO–cGMP–PGK-axis. Drugs targeting the NO–cGMP–PGK-axis aim to promote formation of cGMP, which increases PKG activity. PKG plays a pivotal role in titin phosphorylation contributing to reduction in cardiomyocyte passive stiffness [136]. PKG phosphorylation targets can also lower levels of key transcription factors and sarcomeric proteins mediating LV hypertrophy, diastolic relaxation, LV stiffness, and vasorelaxation. Furthermore, PKG-dependent phosphorylation of phospholamban can improve sarcoplasmic reticulum Ca^2+^-ATPase (SERCA) activity [137] and, therefore, helps to prevent Ca^2+^ mishandling. PDE5 inhibitors (I) protect cGMP from degradation by PDE5. While sGC activators (II) bind to nonoxidized sGC (Fe^2+^), sGC stimulators (III) target oxidized sGC (Fe^3+^). Neprilysin inhibitors (V) prevent degradation of natriuretic peptides, particularly ANP and BNP, which can then bind to pGC. NO-donating drugs (IV) enhance bioavailability of NO, leading to stimulation of sGC. By binding to β3-AR on endothelial cells, β3-AR-selective agonists (VI) promote activity of eNOS, resulting in production of NO. The eNOS enhancer AVE3085 (VII) directly affects eNOS. *ANP* atrial natriuretic peptide, *β3-AR* β3 adrenergic receptor, *BH2* dihydrobiopterin, *BH4* tetrahydrobiopterin, *BNP* B-type natriuretic peptide, *DHFR* dihydrofolate reductase, *DPP4* dipeptidyl peptidase-4, *eNOS* endothelial nitric oxide synthase, *GTP* guanosine triphosphate, *PDE* phosphodiesterase, *pGC* particulate guanylate cyclase, *PKG* protein kinase G, *ROS* reactive oxygen species, *sGC* soluble guanylate cyclase. Figure modified according to Papp et al. [138] and Kovacs et al. [139]

Intervention in the nitrogen monoxide–cyclic guanosine monophosphate–protein kinase (NO–cGMP–PKG)-axis represents a new promising approach in treatment of HFpEF. Experimental data suggest that disturbance of this signal cascade poses a specific pathomechanism in HFpEF, which promotes myocardial fibrosis, eventually leading to diastolic dysfunction [87, 88]. Therefore, targeting the NO–cGMP–PKG pathway with phosphodiesterase-5 (PDE5) inhibitors, soluble guanylyl cyclase activators/stimulators, angiotensin receptor neprilysin inhibitor as well as NO-inducing drugs such as organic nitrates, inorganic nitrites/nitrates, β_3_ adrenergic receptor (β_3_-AR)-selective agonists, or endothelial nitric oxide synthase (eNOS) enhancer have been studied (Fig. 3).

##### Enhancing NO bioavailability

*NO-donating drugs* Direct NO donators, for instance organic nitrates (isosorbide-nitrate), are not recommended in HFpEF patients. In the multicenter trial Neat-HFpEF on 110 patients with HFpEF, isosorbide mononitrate treatment even resulted in decreased activity levels [89]. One major disadvantage of organic nitrates is a strong vasodilatation, which can reduce systemic blood pressure dramatically. Inorganic nitrites, on the other hand, appear to improve ventricular performance with stress, especially by reducing pulmonary capillary wedge pressure (PCWP) and bear a much lower risk for reduction of systemic blood pressure [45]. Moreover, inorganic nitrites evolve their specific effects on hemodynamics precisely during exercise, presumably when patients benefit the most from symptom relief [90]. These effects also account for inorganic nitrates, the precursor to nitrite [91]. However, in the multicenter RCT INDIE-HFpEF, treatment with inhaled inorganic nitrite failed to increase exercise capacity, QOL, NYHA functional class, diastolic function (*E*/*e*′), and NT-proBNP levels [92]. As for now, results of the ongoing KNO3CKOUT-HFpEF trial, investigating effects of orally active potassium nitrate capsules, should be awaited, as they could differ from these previous findings (NCT02840799).

*β*_*3*_*AR-selective agonists* Conventional beta-blockers mainly target β_1_- and β_2_-adrenoreceptors (β_1_-AR/β_2_-AR), which can mediate maladaptive effects of prolonged catecholamine exposure including cardiac remodeling [93]. Moreover, a third subtype of β-adrenoreceptors, β_3_-AR, has been identified in human hearts [94]. In contrast to β_1_-AR and β_2_-AR, these receptors prevent the myocardial hypertrophic response to neurohormonal stimulation [95]. As a result, the concept of stimulating β_3_-AR with the selective agonist mirabegron as a therapeutic option in HFpEF is currently studied in two clinical trials (NCT02775539, NCT02599480).

*Endothelial nitric oxide synthase (eNOS) activators* Enhancing eNOS activity by the transcription amplifier AVE3085 results in increased production of NO and was shown to be associated with a significant improvement in diastolic function in a rat model [96]. However, clinical evaluation of the approach is still pending.

*Potential limitations of enhancing NO bioavailability* According to a recent mouse model, nitrosative stress needs to be acknowledged as one of the main drivers in HFpEF rather than the limited bioavailability of NO [97]. In this model, concomitant metabolic and hypertensive stress resulted in increased activity of inducible nitric oxide synthase (iNOS) which interfered with the inositol-requiring protein 1α (IRE1α)—X-box-binding protein 1 (XBP1) pathway. These findings could explain why NO-inducing approaches have failed so far and could lead to new approaches targeting nitrosative stress, particularly inhibition of iNOS activity, in the future.

*Phosphodiesterase-5 inhibitors* Therapy with the PDE5 inhibitor sildenafil did not improve p*V*O_2_ in HFpEF patients without evidence of pulmonary hypertension (PH) [98] and failed to significantly lower pulmonary artery pressure (PAP) and to improve hemodynamic parameters in patients suffering from HFpEF and resulting postcapillary PH [99]. However, use of sildenafil is an established therapy regimen in patients with precapillary PH and may be considered in certain forms of combined pre- and postcapillary PH (CpC-PH) when coexistence of pulmonary arterial hypertension (PAH) and left heart disease is most likely. In accordance, it was shown that sildenafil can yield positive therapeutic effects in patients with HFpEF and severe forms of CpC-PH [100]. Translation of these findings into general therapeutic recommendations needs to be evaluated in future studies (2010-020153-14).

*Soluble guanylyl cyclase stimulators and activators* Vericiguat and riociguat, primarily used to treat PH, have been analyzed in HF patients in phase 2 clinical studies. As the SOCRATES-PRESERVED trial has shown, vericiguat improved QOL, but failed to reduce NT-proBNP levels or left-atrial volumes [101]. Currently, therapy with sGC stimulators and activators is further studied in various trials (NCT03153111, NCT03254485, NCT02744339, and NCT03547583). The RCT VITALITY-HFpEF (NCT03547583) for instance, will primarily evaluate treatment effects of vericiguat regarding physical function assessed by the KCCQ PLS (Kansas City Cardiomyopathy Questionnaire Physical limitation score).

##### Anti-diabetic drugs

*Sodium-glucose cotransporter-2 inhibitors* After empagliflozin led to a striking reduction of CV events in patients with type 2 diabetes at high CV risk in the EMPA-REG OUTCOME study [102], treatment with SGLT2 inhibitors was evaluated in HF patients with and without diabetes. As shown in the recent DAPA-HF trial, dapagliflozin resulted in a significant decrease of the primary composite endpoint of worsening HF or CV death in 4744 HFrEF patients, regardless of the presence or absence of diabetes [103]. Among the various pathomechanisms under discussion are the increase in renal function due to inhibition of the tubuloglomerular feedback system, the reduction in heart load as a result of the decrease in preload and afterload, and the improvement in cardiac energetics through an increase in ketones’ supply [104, 105]. However, it is unknown whether these effects will account for HFpEF patients also. Finally, experimental data suggested that empagliflozin causes direct pleiotropic effects by improving diastolic stiffness, which are independent of diabetic conditions [106]. Currently, two large phase-III RCTs, including both HFpEF patients with and without diabetes, will investigate effects of the SGLT2 inhibitors empagliflozin (EMPEROR-PRESERVED; NCT03057951) and dapagliflozin (DELIVER; NCT03619213) on HF hospitalizations and CV mortality. In addition, the PRESERVED-HF trial with dapagliflozin (NCT03030235) and the EMPERIAL-PRESERVED trial with empagliflozin (NCT03448406) will primarily focus on treatment effects in regard to exercise capacity as measured by the 6MWD and NT-pro-BNP levels. According to a recent press release, empagliflozin did not have any significant effects on the primary endpoint in the EMPERIAL-PRESERVED trial [107].

*Incretins* Modulation of the incretin system includes mimicking glucagon-like peptide 1 (GLP-1) effects and inhibition of the GLP-1-degrading enzyme dipeptidyl peptidase-4 (DPP-IV) [108]. GLP-1, one of the major incretins, is released after food intake and stimulates insulin secretion from pancreatic β-cells [108]. The corresponding GLP-1 receptors are also found in cardiac myocytes and in certain regions of the brain [109]. In large cohorts of patients with type 2 diabetes at high CV risk, semaglutide and liraglutide, both GLP-1 mimetics, were able to significantly reduce mortality [110, 111]. Currently, there is only one small trial in HFpEF patients, which investigates effects of sitagliptin on hemodynamics as well as diastolic dysfunction and LV hypertrophy (NCT-2012–002,877-71).

##### Targeting cardiac fibrosis and inflammation

*Pirfenidone* represents an anti-fibrotic drug which targets the TGF-β signaling pathway and is mainly used in idiopathic pulmonary fibrosis [112]. By activation of myofibroblasts, TGF-β can promote the production of fibronectin, proteoglycans and type I–III collagen. In mouse models with pressure-overload induced HF, pirfenidone inhibits progression of contractile dysfunction and LV fibrosis after beginning of treatment [113]. The PIROUETTE-trial will investigate whether these effects account for HFpEF patients also (NCT02932566).

*Cross-link breakers* Cross-link breakers target advanced glycation endproducts (AGEs), which are formed by proteins and carbohydrates that underwent “cross-linking” with the extracellular matrix [114]. Production of AGEs is triggered by oxidative stress and is associated with impaired diastolic function [115]. In a small cohort of 23 patients, treatment with the cross-link breaker Alagebrium chloride decreased LV mass and improved LV diastolic filling and QOL [116]. A similar concept which aims to interfere with the formation of AGEs is the antibody-mediated inhibition of the enzyme Lysyl oxidase-like 2 (Loxl2). Loxl2 can contribute to the cross-linking of collagen, eventually leading to interstitial fibrosis and diastolic dysfunction [115]. In mouse models, inhibition of Loxl2 improved systolic and diastolic function [117]. Clinical evaluations of Loxl2-inhibition and new cross-linking strategies have to be awaited.

*Micro-RNAs* Micro-RNAs (miRNAs) are small non-coding RNA molecules which can interfere with gene expression on a post-transcriptional level by binding to messenger-RNA [118]. There is a variety of different miRNAs, and their profiles typically differ between patients with HFpEF and HFrEF [119, 120]. For instance, inhibition of miRNA-21 prevented development of HFpEF, which was associated with reduced expression of the anti-apoptotic gene Bcl-2 in rats [121]. Therefore, targeting miRNAs and trying to interfere with their effects might introduce a new potential therapy regimen in the future. However, the knowledge about the mechanisms of action is incompletely resolved and needs to be understood better before these concepts will be tested in clinical trials.

*Cytokine inhibitors* Derived from the pathophysiological model of systemic inflammation being one of the main mediators in the development of HFpEF, cytokine inhibitors have been tested as therapeutic options. In the D-HART2 trial [122], interleukin-1 (IL-1) blockade with anakinra was not able to improve aerobic exercise capacity in terms of *V*O_2_ and ventilatory efficiency. However, in a sub-analysis of the large RCT CANTOS [123] including patients with previous myocardial infarction, increased high-sensitivity C-reactive protein levels and history of HF, therapy with canakinumab, a monoclonal antibody targeting IL-1ß, significantly decreased risk of HF hospitalizations as well as the composite of HF hospitalization or HF-related mortality [124].

*Cell therapy* Cell therapy targets myocardial inflammation and myocardial fibrosis in HFpEF. In rat models, application of cardiosphere-derived cells (CDCs) decreased LV fibrosis and inflammatory infiltrates achieving normalization of LV relaxation and diastolic pressures and, therefore, led to an improvement in survival [125]. The safety of this concept will be studied in the ongoing REGRESS-HFpEF-trial (NCT02941705). Furthermore, a pilot study on 14 patients with HFpEF showed that treatment with CD34^+^ cells, collected by apheresis after G-CSF stimulation, resulted in an enhancement in diastolic function (*E*/*e*′), and decreased NT-proBNP levels [126]. CD34^+^ cell therapy in patients with HFpEF is currently under further evaluation in the CELL-pEF-trial (NCT02923609). However, cell therapy has been evaluated as a promising therapy for CV diseases in numerous past trials without delivering consistent and convincing results. Questions about optimal cell type, dose, and delivery route are still inadequately answered [127].

##### Restoring mitochondrial energy

*Szeto-Schiller peptides* “Szeto-Schiller peptides (SS peptides)” belong to a new class of antioxidant peptides that bind to the cardiolipin, an important phospholipid in the inner mitochondrial membrane. SS peptides protect cardiolipin from oxidation and, thereby, prevent the damage of oxidative stress to mitochondria, maintaining ATP production and reducing further oxidative stress [128]. The most prominent and first compound is elamipretide (MTP-131, SS31) which has been subject of clinical studies after experimental data delivered encouraging results [129]. In the EMBRACE-STEMI study, elamipretide was safe, but failed to reduce infarct size as assessed by CK-MB levels in patients during/after ST-elevation myocardial infarction and successful percutaneous coronary intervention [130]. In patients with HFpEF, elamipretide reduced left-ventricular end-diastolic volumes compared to placebo after 4 h of infusion [131]. Currently, two phase II clinical trials test the clinical efficacy of elamipretide in patients with acute or chronic HFpEF (NCT02814097, NCT02914665).

*Adenosine A1 receptor agonists* The partial adenosine A1 receptor agonist Neladenoson bialanate is thought to yield several beneficial effects in the heart and also in the skeletal muscle. These compromise improvement in mitochondrial function and energy substrate utilization, enhanced SERCA2a activity, reversion of ventricular remodeling, and providing anti-ischemic properties [132]. In the PANACHE-trial (NCT03098979), treatment with Neladenoson bialanate failed to significantly affect the primary endpoint “change in 6MWD” in HFpEF patients [133].

##### Targeting intracellular calcium homoeostasis and calcium sensitivity

*Levosimendan* According to ESC guidelines, levosimendan, a calcium sensitizer and PDE3 inhibitor with vasodilative properties [104], can be considered in patients with acute HF and severe reduction of cardiac output (CO), resulting in compromised vital organ perfusion [7]. The positive inotropic effect of levosimendan is the result of a combined effect on troponin C (sensitization to calcium binding) and PDE3-inhibition, increasing cAMP and calcium [104]. Moreover, infusions of levosimendan decreased PAP, NT-proBNP levels, and inflammatory status by altering the ratio of interleukin-6 to interleukin-10 as well as improved diastolic function and right-ventricular systolic function in 54 patients with advanced HF due to left heart failure (NYHA III–IV, LVEF < 35%) [134]. Thus, the current RCT HELP will investigate the effects of levosimendan in 36 HFpEF patients with diagnosed group 2 PH (PH due to left heart disease) (NCT03541603).

##### Prostacyclin analogues

In patients with group 2 PH and HFpEF, administration of inhaled iloprost led to an acute reduction of PAP and pulmonary vascular resistance (PVR) during right heart catheterization [135]. The two RCTs ILO-HOPE and SOUTHPAW will help to further evaluate treatment effects of prostacyclin analogues in patients suffering from HFpEF (NCT03037580, NCT03620526).

#### Device therapies

##### Home monitoring

Fluid overload in patients with HFpEF can rapidly reduce QOL by causing dyspnea and peripheral edema or even lead to hospitalization due to cardiac decompensation. Since hospitalization for HF is associated with a higher mortality risk [136], monitoring of HF patients to avoid symptom deterioration or hospitalization has come into focus as one important part of the therapy. In 2007, the first studies introduced monitoring of HF patients with a new radiofrequency-based wireless pressure sensor (CardioMEMS device), implanted into the pulmonary artery and which continuously monitors pulmonary artery pressure [137, 138]. In the subsequent CHAMPION trial, usage of CardioMEMS in NYHA III patients with HFpEF and HFrEF was able to reduce HF-related hospitalizations [139]. The 1 year outcome results of the CardioMEMS Postapproval study confirmed efficacy of home monitoring in 1200 HF patients [140]. After implantation of the CardioMEMS device, event rate of HF hospitalization/all-cause death per patient year was reduced by 44%. The large multicenter RCT GUIDE-HF will try to reproduce these results in 3600 symptomatic HF patients (NCT03387813).

##### Atrial shunt device

The idea of creating artificial left–right shunts to reduce left-atrial pressure originates from a publication from the early twentieth century. The so-called Lutembacher syndrome was used to describe the finding that patients with an untreated mitral stenosis and resulting increase in left-atrial pressure benefit from a concomitant atrial septal defect [141]. In a pilot trial, which included 11 patients with an LVEF of at least 45%, implantation of an atrial shunt device led to a significant reduction of LV-filling pressures after 30 days [142]. Remarkably, no patient developed PH after the procedure. Analogously, the REDUCE LAP-HF I trial on a total of 64 patients recorded a reduction in left-atrial pressure during exercise with improvement in functional capacity and QOL after shunt implantation [143]. The latest 1-year results of REDUCE LAP-HF I showed no significant differences in major adverse cardiac, cerebrovascular, or renal events compared to patients who underwent sham procedure, suggesting this method to be safe [144]. In the future, the REDUCE-LAP-HF II trial, which focuses on clinical outcomes, will hopefully take up from these positive results (NCT03088033).

##### Cardiac resynchronization therapy (CRT)

In HFpEF patients, LV mechanical dyssynchrony has been suggested to contribute to an impairment of longitudinal systolic and diastolic LV function and to be associated with higher LV-filling pressures and worse clinical status in terms of NYHA functional class [145]. On the other hand, a post hoc analysis of the TOPCAT trial has shown that LV mechanical dyssynchrony is not associated with outcomes of HFpEF patients [146]. Targeting LV dyssynchrony by implantation of a CRT device is currently subject of ongoing studies, which will help to better understand its relevance for the therapy of HFpEF patients (NCT03338374, NCT02145351).

##### Cardiac contractility modulation (CCM)

CCM aims to trigger molecular remodeling by delivering electrical signals into the septum during the refractory period and has been associated with numerous beneficial effects in chronic HF such as increased titin and troponin phosphorylation, and reduced expression of proteins that mark cardiac fibrosis [147]. In two female patients, CCM has been shown to improve clinical status and echocardiographic parameters early after initiation of CCM therapy [148]. The CCM-HFpEF trial will study the effects of CCM on QOL in patients with HFpEF (NCT03240237).

##### Renal denervation

Renal denervation (RDN), a catheter-based, radiofrequency ablation of the renal sympathetic nerves, has been shown to effectively lower both systolic and diastolic blood pressure [149, 150]. Furthermore, reduction of cardiac sympathetic activity occurs independently from blood pressure reduction, suggesting direct effects on the heart [151]. As a consequence, RDN reduced LV mass and improved diastolic function [152–154]. However, the underpowered RDT-PEF trial including 25 patients with HFpEF did not confirm a beneficial effect of RDN on diastolic parameters and QOL [155]. Further investigations are needed to clarify the therapeutic value of RDN in HFpEF.

### What is left?

Fluid overload can cause preload increase and as a result cardiac decompensation. Patients may suffer from peripheral edema and signs of congestion such as dyspnea. Therefore, diuretics, which are established drugs to treat fluid overload and signs of congestion, are a cornerstone in the symptomatic therapy of HFpEF [7]. Treatment with ACE inhibitors or ARBs did not result in further improvement regarding QOL, exercise capacity, or myocardial function after initiation of optimal diuretic therapy [156]. However, overtreatment should be avoided as it can cause excessive preload reduction and reduction in filling pressures, which stiff hearts may depend on [33].

### Summary and perspective

The 2016 ESC/HFA guidelines [7] acknowledge the fact that no treatment has been convincingly shown to reduce morbidity or mortality in HFpEF patients. Since then, further efforts have been made to improve understanding and treatment of the HFpEF syndrome. However, diagnosis of HFpEF remains controversial and there is growing appreciation that HF, and particular HFpEF, represents a heterogeneous syndrome with various phenotypes and comorbidities which are hardly to differentiate solely by LVEF and might benefit from individually tailored approaches [9, 157]. These aspects are also supported by the results of the recently presented PARAGON-HF trial [53], which failed to show beneficial treatment effects of LCZ696 in HFpEF patients, but has been associated with positive effects in female patients and patients with an LVEF between 45–57%. In the future, prospective randomized studies should be conducted in well-defined, dedicated subgroups which take various information (clinical characteristics, biomarker levels, and imaging modalities) into account. In this context, new diagnostic techniques such as novel imaging strategies may help to differentiate the etiologies of HFpEF, to identify situations with specific treatment options, and to stratify available treatments. Among the various therapeutic approaches that have been introduced lately, therapy with SGLT2 inhibitors promises the greatest potential for the future, as it has just been proven efficient in HFrEF patients and is currently studied in two large RCT. In addition, innovative device therapies, in particular creating artificial left–right shunts by implantation of an atrial shunt device, pose exciting options for the future, but need to be proven safe first. As for now, treatment of HFpEF is limited to symptom relief, which effectively improves QOL. Therefore, further research is desperately needed, to manage the challenging syndrome HFpEF.
